# Optimal Cut-off Points of the Standardized Continuous Metabolic Syndrome Severity Score (cMetS-S) for Predicting Cardiovascular Disease (CVD) and CVD Mortality in the Tehran Lipid and Glucose Study (TLGS)

**DOI:** 10.5812/ijem-154255

**Published:** 2024-12-18

**Authors:** Maryam Adib, Ladan Mehran, Safdar Masoumi, Iman Vatanpoor, Fereidoun Azizi, Atieh Amouzegar

**Affiliations:** 1 Endocrine Research Center, Research Institute for Endocrine Sciences, Shahid Beheshti University of Medical Sciences, Tehran, Iran; 2 Department of Cellular and Physiological Sciences, University of British Columbia, Vancouver, Canada

**Keywords:** Continuous Metabolic Syndrome Severity Score, Cardiovascular Disease, Mortality, Metabolic Syndrome

## Abstract

**Background:**

Metabolic Syndrome (MetS) is a prevalent condition associated with an increased risk of cardiovascular disease (CVD) and CVD mortality. Due to the limited clinical applicability of MetS, the standardized continuous metabolic syndrome severity score (cMetS-S) has the potential to provide continuous assessment of metabolic risk.

**Objectives:**

This study evaluated the optimal cMetS-S cut-off points in the Tehran Lipid and Glucose Study (TLGS) for predicting CVD and CVD mortality.

**Methods:**

The study included 7,776 participants over 30 years old at baseline, followed for 18 years. Sex-specific sensitivity (SS) and specificity (SP) of cMetS-S measures for predicting CVD and CVD mortality were evaluated using a receiver operating characteristic (ROC) curve, along with the area under the curve (AUC), employing a naive estimator and considering event failure status and MetS variables.

**Results:**

The cut-off point of cMetS-S for CVD was 0.13 (SS: 65.5%, SP: 59.6%) for the total population, 0.44 (SS: 49.6%, SP: 68.1%) for men, and 0.27 (SS: 64.2%, SP: 69.2%) for women. The cut-off point of cMetS-S for CVD mortality was 0.53 (SS: 51.3%, SP: 71.9%) for the total population, 0.76 (SS: 35.1%, SP: 76.2%) for men, and 0.28 (SS: 78.8%, SP: 66.4%) for women. The AUC (95% CI) of MetS based on the International Diabetes Federation (IDF) and Joint Interim Statement (JIS) definitions were 60.0 (65.3 - 56.8) and 61.1 (59.6 - 56.8) for CVD, and 59.3 (56.0 - 62.5) and 59.4 (56.3 - 62.6) for CVD mortality.

**Conclusions:**

The cut-off points of cMetS-S for CVD and CVD mortality differ between men and women. The cMetS-S could be a better predictive tool for CVD and CVD mortality than MetS.

## 1. Background

Metabolic syndrome (MetS), a group of metabolic abnormalities, is a common condition linked to the risk of cardiovascular disease (CVD) and CVD mortality, creating a significant socioeconomic burden globally (1-4). The prevalence of MetS in Iranian adults exceeds 30% (5-7), which is higher than the global prevalence (8). The binary definition of MetS (presence/absence) limits its application in clinical settings (6). An important drawback of the binary MetS definition is that the severity of the disease is overlooked, and slight alterations in the value of each MetS component may inaccurately label people as having MetS or not (6, 9).

To overcome these constraints, a few scientists have calculated a continuous MetS severity score (cMetS-S) using confirmatory factor analysis (CFA), considering the weighted contribution of MetS components and their variations based on age, sex, and ethnicity (10-12). Recently, it has been shown that cMetS-S has better clinical utility than conventional MetS criteria for predicting cardiovascular events and CVD mortality (6, 13-16). The cMetS-S has been developed for the Iranian population (10), and a previous study showed its association with CVD events with a hazard ratio (HR) of 1.67 (95% CI: 1.47 - 1.89) upon an increase of 1 standard deviation (SD) in cMetS-S (6). The standardized cMetS-S has the potential to provide a more nuanced and continuous assessment of metabolic risk and could potentially be introduced as a new global scoring system for MetS. It can better stratify patients into risk categories for CVD and CVD mortality based on severity rather than just the presence or absence of MetS, and help track disease progression and treatment effectiveness.

The clinical applicability of MetS is limited due to its discontinuous and categorical nature. In contrast, the standardized cMetS-S has the potential to provide a more nuanced and continuous assessment of metabolic risk. By capturing the gradual progression of metabolic abnormalities, cMetS-S may offer a more comprehensive prediction of CVD and CVD mortality compared to traditional MetS categorization.

## 2. Objectives

This study evaluated the optimal cMetS-S cut-off points that indicate the best predictive power for CVD and CVD mortality, and compared the predictive power of cMetS-S and MetS for CVD and CVD mortality.

## 3. Methods

### 3.1. Study Population

**Figure 1.**
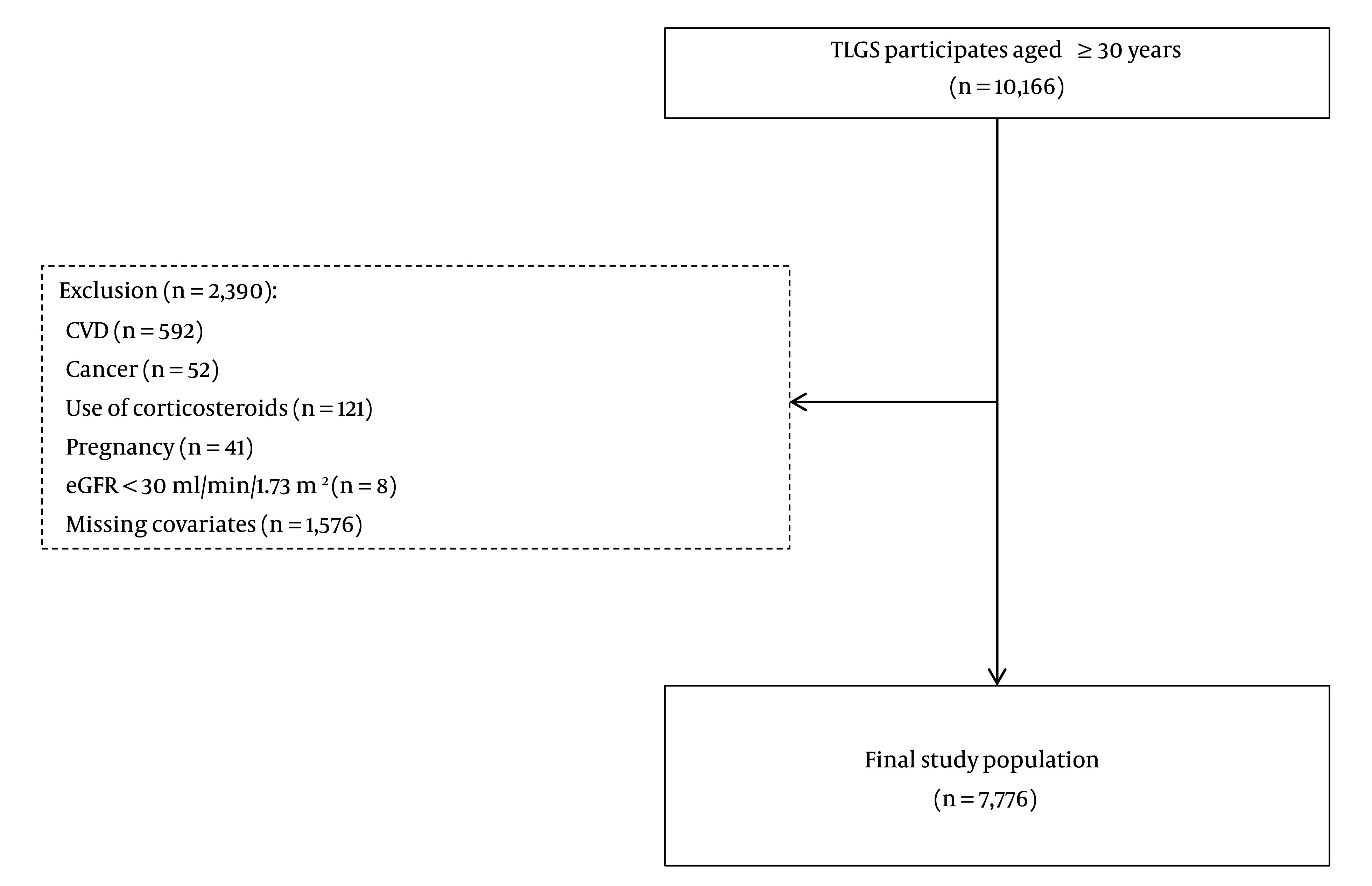
Flowchart illustrating participants selection. CVD, cardiovascular disease; TLGS, Tehran Lipid and Glucose Study; eGFR, estimated glomerular filtration rate

Participants were recruited from the Tehran Lipid and Glucose Study (TLGS), a large 20-year cohort study initiated in 1999 in Iran. The TLGS aimed to determine the prevalence, risk factors, and health outcomes of non-communicable diseases in a representative sample of the Iranian population. The study’s design has been detailed in another publication (17). In this study, we enrolled individuals from phase I and new entries for phase II of the TLGS study who were older than 30 years at baseline and were followed up every 3 years for 18 years, from 1999 to 2018. Participants with cancer (n = 52), CVD (n = 592), estimated glomerular filtration rate (eGFR) less than 30 mL/min/1.73 m² (n = 8), use of systemic corticosteroids (n = 121), pregnancy (n = 41), or missing covariates (n = 1576) at baseline were excluded. Finally, a total of 7776 participants entered the study (Figure 1). 

### 3.2. Measurements and Definitions

The trained physician collected demographic data using questionnaires, including age, sex, marital status, physical activity, educational level, past medical and medication history, and smoking habits. Participants were divided into three groups based on physical activity. Information of physical activity for individuals entering phase I was gathered using the Persian version of the Lipid Research Clinic (LRC) questionnaire. For those newly entering phase II, the Metabolic Equivalent of Task Scale (METS) was used. Participants with METS < 600 min/week were classified as having low-level physical activity, 600 < METS < 3000 min/week were classified as having moderate physical activity, and METS > 3000 min/week were classified as having high physical activity. Educational levels were categorized into three groups: (1) Less than 6 years of schooling, considered illiterate or elementary education, (2) 6 - 12 years of schooling, considered secondary education; and (3) more than 12 years of schooling, considered higher education. Smoking status was classified into two categories: Individuals who used tobacco products occasionally or daily in the past month were considered current smokers, and those who had not used tobacco products in the past month or ever were considered non-smokers. A positive family history of premature CVD was defined as a diagnosis of CVD by a physician in at least one first-degree relative under 65 years of age in women and 55 years of age in men. Height, waist circumference (WC), and weight were measured while participants were minimally clothed and without shoes, using standard protocols, with measurements rounded to the nearest 0.1 cm and 0.1 kg using a tape measure and a portable digital scale. Body Mass Index (BMI) was determined using the following equation: Weight (kg)/height^2^ (m). Following 15 minutes of sitting in a resting position, two measurements of systolic blood pressure (SBP) and diastolic blood pressure (DBP) on the right arm were taken using a standardized mercury sphygmomanometer (adjusted by the Iranian Institute of Standards and Industrial Research); the average of the two measurements was recorded as the participant’s blood pressure (BP). Systolic blood pressure ≥ 140 mmHg and/or DBP ≥ 90 mmHg, or the use of antihypertensive drugs, were considered as hypertension. Participants’ venous blood samples were collected between 7 - 9 AM after 12 - 14 hours of overnight fasting and were tested in the TLGS research laboratory on the same day they were collected. Enzymatic colorimetric methods were used to measure triglycerides (TG), high-density lipoprotein cholesterol (HDL-C), total cholesterol (TC), and fasting blood sugar (FBS) levels. The criteria for dyslipidemia included TC ≥ 200 mg/dL, TG ≥ 150 mg/dL, HDL-C ≤ 40 mg/dL in men and HDL-C ≤ 50 mg/dL in women, or the use of lipid-lowering drugs. The kinetic colorimetric Jaffe method (Pars Azmoon kit, Tehran, Iran) was used to measure the creatinine level. The chronic kidney disease epidemiology collaboration (CKD-EPI) 2021 formula was used to calculate the eGFR (18).

### 3.3. Definition

- The cMetS-S: Age and sex-specific cMetS-S is a novel metric that has previously been developed and validated from the TLGS, aimed at assessing the severity of MetS in adults aged 20 - 60 (10). The equations derived from CFA, considering the weight of each MetS component based on sex and age classification (Appendix 1 in Supplementary File), indicate that higher cMetS-S values reflect greater MetS severity (10). In this study, it was standardized to a mean of 0 and a SD of 1 according to the study population for easier interpretation and to improve the generalizability of the results (10).

- Cardiovascular disease: In this study, CVD is defined as the presence of any component of coronary heart disease (CHD), stroke, or vascular-related mortality. Components of CHD include: (1) Definitive myocardial infarction, based on positive findings in the electrocardiogram (ECG) and positive biomarkers; (2) possible myocardial infarction, based on positive findings in the ECG accompanied by cardiac symptoms and negative or borderline biomarkers, or positive ECG findings along with borderline biomarkers; (3) unstable angina, characterized by new cardiac manifestations or a change in the pattern of cardiac symptoms, with positive ECG findings and negative biomarkers; (4) proven CHD through angiography; (5) CHD-related mortality, including death based on the above criteria or sudden cardiac death occurring within one hour of symptom onset. Also, stroke is defined as either definitive or possible stroke. Possible stroke is characterized by new neurological deficits lasting longer than 24 hours (19, 20).

- International Diabetes Federation (IDF): The IDF defines MetS as the presence of central obesity (WC ≥ 94 cm for men and ≥ 80 cm for women) plus any two of the following factors: TG ≥ 150 mg/dL, HDL-C < 40 mg/dL in men and < 50 mg/dL in women, BP ≥ 130/85 mmHg, or FBS ≥ 100 mg/dL (21).

- Joint Interim Statement (JIS): The JIS defines MetS as the presence of central obesity (WC ≥ 94 cm for men and ≥ 80 cm for women) plus any three of the following factors: TG ≥ 150 mg/dL, HDL-C < 40 mg/dL in men and < 50 mg/dL in women, BP ≥ 130/85 mmHg, or FBS ≥ 100 mg/dL (22).

### 3.4. Statistical Analysis

The study population's baseline characteristics were summarized using the mean and SD, with comparisons between men and women made through two-tailed independent *t*-tests. Sensitivity (SS) and specificity (SP) were defined in the context of a standard receiver operating characteristic (ROC) curve, where SS refers to the probability of correctly identifying diseased subjects, and SP refers to accurately identifying non-diseased subjects. A naive estimator was used to compute these metrics, along with the area under the curve (AUC). All statistical analyses, including ROC curves and cut-off evaluations for cMetS-S and components of MetS, were performed using STATA 14.2 (23), with a significance threshold set at a P-value < 0.05. The cut-off for cMetS-S and components of MetS was calculated using the maximum value of the Youden Index = SS + SP - 1, in each men and women group. The cMetS-S and components of MetS for CVD and CVD mortality were assessed by the AUC. Additionally, the predictive power of MetS based on the JIS and IDF definitions for CVD and CVD mortality was compared with cMetS-S (23).

## 4. Results

Table 1 shows the study population's baseline characteristics according to cMetS-S, with separate data for men and women. Appendix 2 in Supplementary File presents the study population's baseline characteristics according to cMetS-S quartiles. Overall, we enrolled 7776 subjects with a mean age of 46.84 ± 12.3 years, of which 43.8% were men. Men were older than women. The average TG levels and prevalence of current smoking were higher in men. However, women had higher mean values of BMI and FBS.

**Table 1.** Study Population’s Baseline Characteristics, According to Metabolic Syndrome Severity Score ^a^

Characteristics	Overall	Men	Women	P-Value
**Number of participants **	7,776	3,403	4,373	
**Age (y)**	46.8 ± 12.3	47.7 ± 13.0	46.2 ± 11.6	< 0.001
**BMI (kg/m** ^**2**^ **)**	27.5 ± 4.5	26.2 ± 3.9	28.52 ± 4.7	< 0.001
**WC (cm)**	90.5 ± 11.4	90.5 ± 10.8	90.60 ± 11.9	< 0.001
**Education**				< 0.001
Illiterate/primary school (< 6 years)	4776 (67.1)	2006 (61.5)	2770 (71.8)	
High school (6 - 12 years)	1428 (20.1)	652 (20.0)	776 (20.1)	
Higher education (> 12 years)	914 (12.8)	601 (18.4)	313 (8.1)	
**Current smoking**	1144 (14.7)	975 (28.7)	169 (3.9)	< 0.001
**Physical activity**				0.004
Low	802 (23.7)	1174 (26.9)	1976 (25.5)	
Moderate	552 (16.3)	708 (16.2)	1260 (16.3)	
High	2026 (59.9)	2477 (56.8)	4503 (58.2)	
**Family history of CVD**	1284 (16.5)	485 (14.3)	799 (18.3)	< 0.001
**SBP (mmHg)**	121.3 ± 19.8	121.4 ± 18.9	121.2 ± 20.4	< 0.001
**DBP (mmHg)**	78.7 ± 10.9	78.4 ± 11.1	78.8 ± 10.7	< 0.001
**FBS (mg/dL)**	100.5 ± 35.0	99.8 ± 31.4	101.1 ± 37.6	< 0.001
**Triglyceride (mg/dL)**	182.3 ± 118.9	192.9 ± 132.5	174.0 ± 106.4	< 0.001
**TC (mg/dL)**	214.4 ± 45.3	207.7 ± 41.9	219.7 ± 47.2	
**HDL-C (mg/dL)**	41.6 ± 10.9	37.8 ± 9.5	44.5 ± 11.1	< 0.001
**Anti-hypertensive drug use**	493 (9.6)	128 (5.8)	365 (12.4)	< 0.001
**Anti-diabetic drug use**	333 (4.3)	116 (3.4)	217 (4.9)	< 0.001
**Lipid-lowering drug use**	209 (2.7)	53 (1.6)	156 (3.6)	< 0.001
**cMetS-S (IQR)**	0.02 (0.28)	0.04 (0.26)	0.0 (0.29)	< 0.001

Abbreviations: BMI, Body Mass Index; CVD, cardiovascular disease; DBP, diastolic blood pressure; SBP, systolic blood pressure; HDL-C, high density lipoprotein cholesterol; FBS, fasting blood sugar; n, number; SD, standard deviation; TC, total cholesterol; WC, waist circumference.

^a^ Values are expressed as No. (%) or mean ± SD unless otherwise indicated.

**Figure 2.**
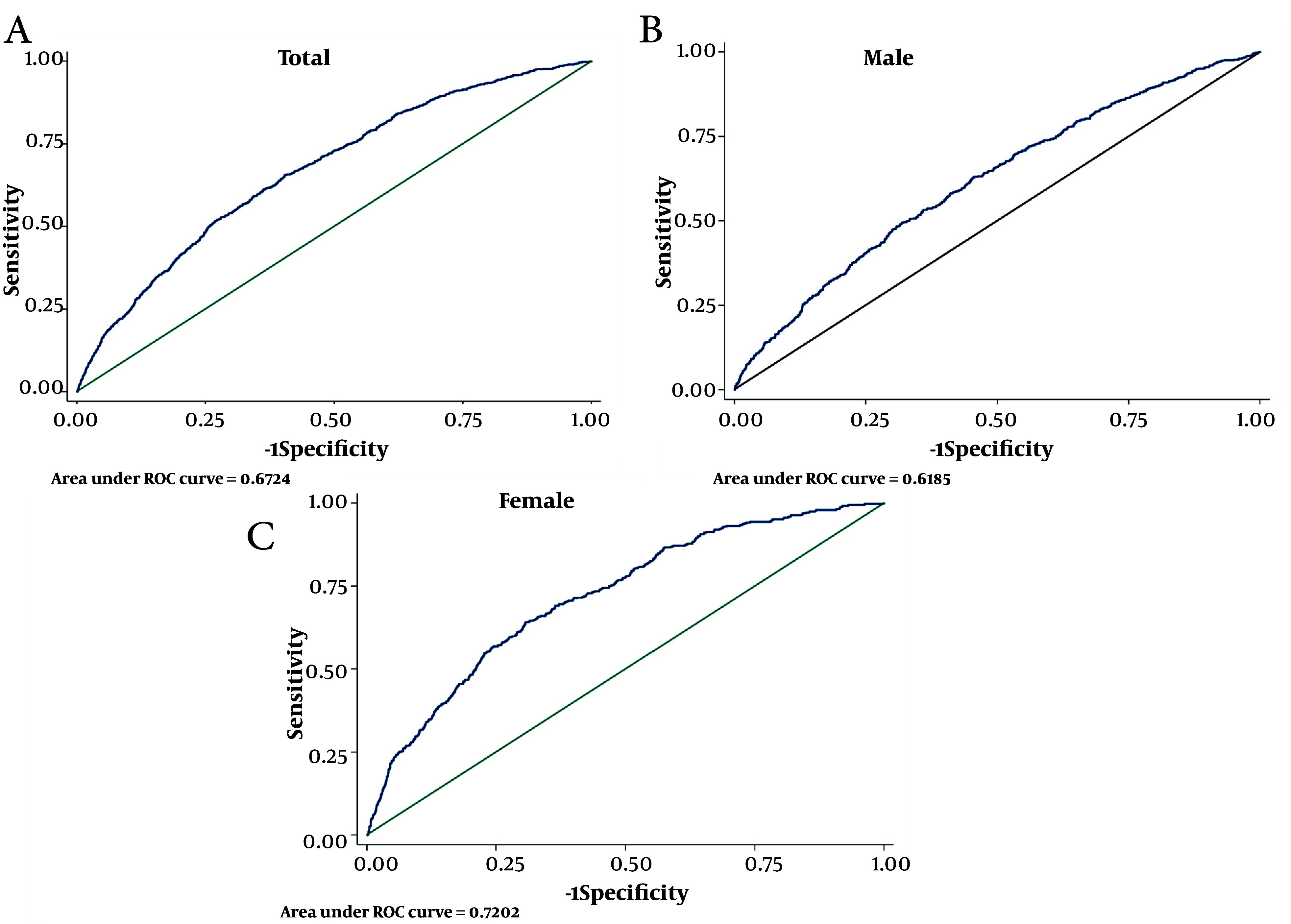
Receiver operating characteristic (ROC) curve metabolic syndrome (MetS) severity score and components metabolic syndrome for incidence cardiovascular disease (CVD)

In this study, the optimal cut-off points for predicting CVD and CVD mortality were determined for cMetS-S and various risk factors of CVD and CVD mortality, including SBP, WC, FBS, and TG. These cut-off points were analyzed for their SS, SP, and Youden Index (Youden-X) in relation to CVD (Table 2) and CVD mortality (Table 3). For the total population, the cMetS-S cut-off point of 0.13 exhibited a SS of 65.5% and SP of 59.6%, with an AUC (95% CI) of 67.2 (65.6 - 68.8) and a Youden-X of 0.25 for CVD; the ROC curve is shown in Figure 2A. For the men subgroups, the cMetS-S cut-off of 0.44 yielded a SS of 49.6% and SP of 68.1%, with an AUC (95% CI) of 61.8 (59.5 - 64.2) and Youden-X of 0.17; the ROC curve is shown in Figure 2B. In the women subgroup, the cMetS-S cut-off of 0.27 showed a SS of 64.2%, SP of 69.2%, AUC (95% CI) of 72.0 (69.7 - 74.3), and Youden-X of 0.33; the ROC curve is shown in Figure 2C. 

**Table 2.** The Sensitivity, Specificity, Area Under the Curve, and Cut-off of Metabolic Syndrome Severity Score and Components Metabolic Syndrome for Incidence Cardiovascular Disease

Variables	SS	SP	AUC	Cut-off	Youden Index
**Total (n = 7776)**					
SBP	61.7	65.3	68.3 (66.7, 70.0)	123	0.27
WC	75.1	42.5	61.8 (60.2, 63.5)	88	0.17
FBS	49.1	72.4	63.8 (62.1, 65.7)	98	0.21
TG	71.3	43.6	60.4 (59.0, 62.1)	139	0.15
Mets JIS	65.3	56.8	61.1 (59.6, 56.8)	-	0.22
Mets IDF	56.4	63.6	60.0 (65.3, 56.8)	-	0.20
CMets-s	65.5	59.6	67.2 (65.6, 68.8)	0.13	0.25
**Men (n = 3403)**					
SBP	61.9	64.2	66.5 (64.1, 68.8)	122	0.26
WC	56.9	55.4	59.3 (56.9, 61.6)	92	0.12
FBS	44.9	71.6	60.5 (58.1, 62.9)	98	0.16
TG	51.0	58.3	55.7 (53.3, 58.1)	177	0.1
Mets JIS	57.9	57.1	61.1 (59.6 ,62.5)	-	0.15
Mets IDF	50.3	63.1	60.0 (58.5, 61.5)	-	0.13
CMets-s	49.6	68.1	61.8 (59.5, 64.2)	0.44	0.17
**Women (n = 4373)**					
SBP	58.6	73.2	71.1 (68.7, 73.5)	128	0.31
WC	77.5	46.9	65.3 (62.9, 67.7)	89	0.24
FBS	53.2	74.8	67.7 (65.0, 70.3)	99	0.28
TG	67.0	54.4	65.0 (62.6, 67.5)	155	0.21
Mets JIS	75.5	56.5	61.1 (59.6, 62.5)	-	0.32
Mets IDF	64.9	63.9	60.0 (58.5, 61.5)	-	0.28
CMets-s	64.2	69.2	72.0 (69.7, 74.3)	0.27	0.33

Abbreviations: SS, sensitivity; SP, specificity; AUC, area under the curve; n, number; DBP, diastolic blood pressure; SBP, systolic blood pressure; WC, waist circumference; TG, triglycerides; FBS, fasting blood sugar; MetS, metabolic syndrome; cMetS-S, continuous metabolic syndrome severity score; IDF, International Diabetes Federation; JIS, joint interim statement.

For the total population, SBP, WC, FBS, and TG cut-off points for CVD were 123, 88, 98, and 139, respectively. The SS, SP, and AUC for these parameters, as well as additional details for men and women subgroups, are provided in Table 2. The AUC (95% CI) of MetS (JIS) for CVD was 61.1 (59.6 - 56.8) with SS: 65.3%, SP: 56.8%, and the corresponding values for MetS (IDF) were 60.0 (65.3 - 56.8) with SS: 56.4%, SP: 63.6% (Table 2). 

**Figure 3.**
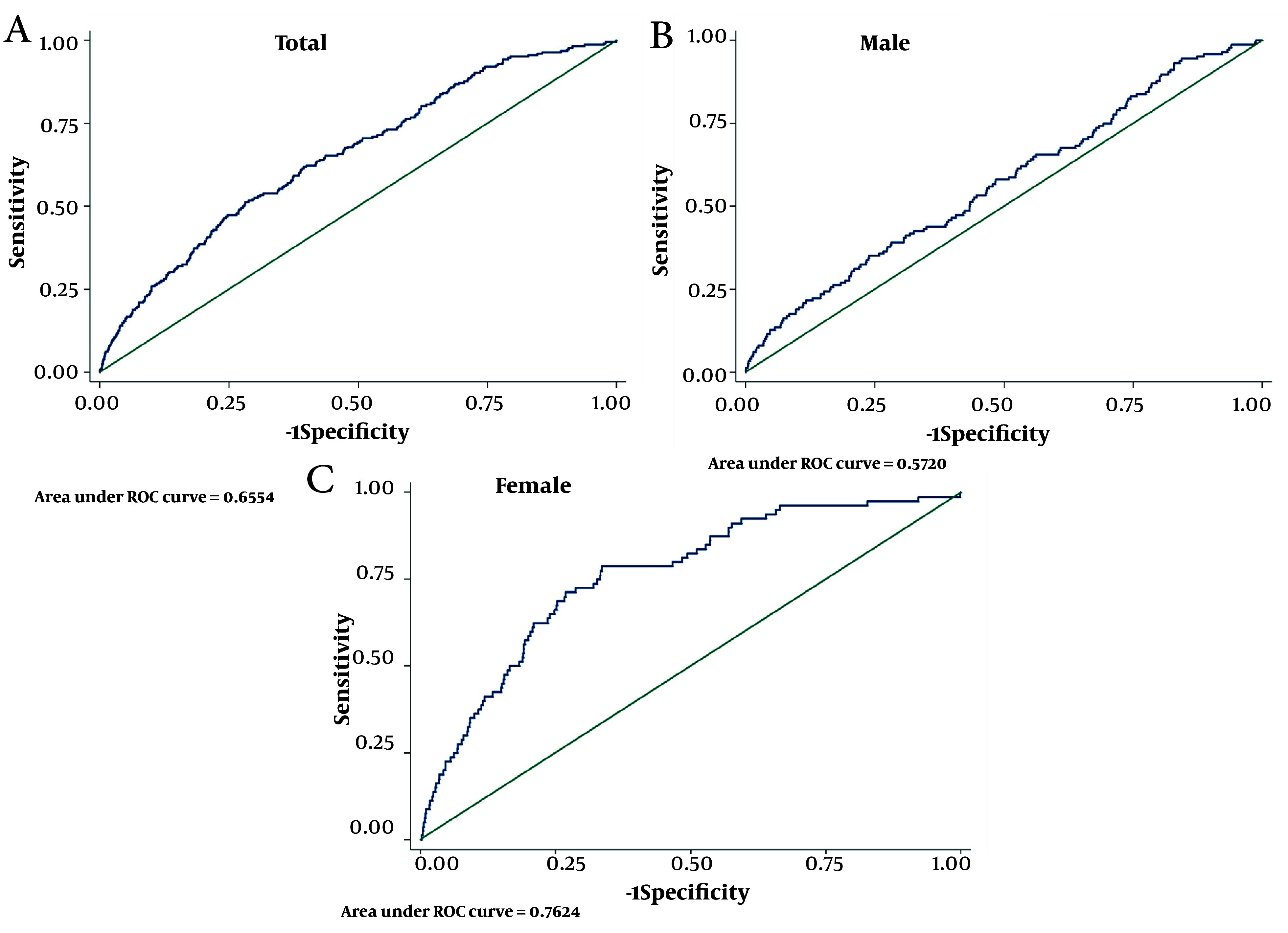
Receiver operating characteristic (ROC) curve metabolic syndrome (MetS) severity score and components metabolic syndrome for cardiovascular disease (CVD) mortality

For the total population, the cMetS-S had a cut-off point of 0.53 with 51.3% SS, 71.9% SP, AUC (95%CI) of 65.5 (62.0 - 69.1), and Youden-X of 0.23 for CVD mortality; the ROC curve is shown in Figure 3A. For the men subgroups, the cMetS-S cut-off point of 0.76 showed a SS of 35.1%, SP of 76.2%, AUC (95%CI) of 57.2 (52.4 - 62.0), and Youden-X of 0.11; the ROC curve is shown in Figure 3B. In the women subgroup, cMetS-S demonstrated a cut-off point of 0.28 with a SS of 78.8%, SP of 66.4%, AUC (95%CI) of 76.2 (71.1 - 81.3), and Youden-X of 0.45; the ROC curve is shown in Figure 3C. 

For the total population, the best cut-off points for SBP, WC, FBS, and TG for CVD mortality were 130, 92, 110, and 170, respectively. The SS, SP, and AUC for these parameters, along with additional details for the men and women subgroups, are presented in Table 3. The AUC (95%CI) of MetS (JIS) for CVD mortality was 59.4 (56.3 - 62.6) with SS: 64.9%, SP: 53.9%, and the corresponding values for MetS (IDF) were 59.3 (56.0 - 62.5) with SS: 57.5%, SP: 61.0% (Table 3). 

**Table 3.** The Sensitivity, Specificity, Area Under the Curve, and Cut-off of Metabolic Syndrome Severity Score and Components Metabolic Syndrome for Cardiovascular Disease Mortality

Variables	SS	SP	AUC	Cut-off	Youden Index
**Total (n = 7776)**					
SBP	64.0	74.1	72.4 (68.9, 76.0)	130	0.38
WC	64.5	53.8	61.4 (57.8, 65.0)	92	0.18
FBS	38.6	86.1	65.8 (61.9, 69.7)	110	0.25
TG	52.6	57.0	55.1 (51.4, 58.7)	170	0.10
Mets JIS	64.9	53.9	59.4 (56.3, 62.6)	-	0.19
Mets IDF	57.5	61.0	59.3 (56.0, 62.5)	-	0.19
CMets-s	51.3	71.9	65.5 (62.0, 69.1)	0.53	0.23
**Men (n = 3403)**					
SBP	54.7	74.9	66.8 (61.9, 71.6)	130	0.30
WC	59.5	53.5	57.8 (53.0, 62.5)	92	0.13
FBS	35.1	88.3	60.9 (55.6, 66.2)	111	0.23
TG	95.3	8.7	47.8 (43.3, 52.3)	78	0.04
Mets JIS	54.7	54.4	54.6 (50.5, 58.7)	-	0.09
Mets IDF	49.3	60.8	55.1 (50.9, 59.2)	-	0.10
CMets-s	35.1	76.2	57.2 (52.4, 62.0)	0.76	0.11
**Women (n = 4373)**					
SBP	81.3	73.4	82.6 (78.1, 87.1)	130	0.55
WC	73.8	54.1	67.9 (62.4, 73.5)	92	0.28
FBS	77.5	58.9	73.8 (68.6, 79.1)	94	0.36
TG	63.8	60.3	65.3 (59.4, 71.3)	172	0.24
Mets JIS	83.8	53.5	68.6 (64.5, 72.8)	1	0.37
Mets IDF	72.5	61.2	66.9 (61.9, 71.8)	1	0.34
CMets-s	78.8	66.4	76.2 (71.1, 81.3)	0.28	0.45

Abbreviations: SS, sensitivity; SP, specificity; AUC, area under the curve; n, number; SBP, systolic blood pressure; DBP, diastolic blood pressure; WC, waist circumference; FBS, fasting blood sugar; TG, triglycerides; MetS, metabolic syndrome; cMetS-S, continuous metabolic syndrome severity score; JIS, joint interim statement; IDF, International Diabetes Federation.

## 5. Discussion

The present study determined the optimal cut-off points of cMetS-S for predicting CVD and CVD mortality in a large, representative sample of Iranian adults during an 18-year follow-up in a population-based cohort study. The cMetS-S cut-off points of 0.13 (SS: 65.5%, SP: 59.6%, AUC: 67.2%) and 0.53 (SS: 51.3%, SP: 71.9%, AUC: 65.5%) were found to be the most appropriate for predicting the risk of CVD and CVD mortality, respectively, and these thresholds were lower in women than in men. Given that the AUC for CVD and CVD mortality was higher for cMetS-S compared to the JIS and IDF's definition of MetS, cMetS-S could be a better predictive tool for CVD and CVD mortality than MetS.

The traditional MetS, characterized by having at least three abnormal MetS components, cannot assess the risk in patients with various combinations of components (10). It was challenging to classify participants whose biological measurement test values were at the threshold of the defined criteria (10). In other words, it remains uncertain whether individuals exhibiting a triad of components face a significantly elevated risk of CVD compared to those with just a dyad of components. Although MetS is a predictor of CVD, its binary criteria cannot determine the importance and severity of its components. This definition is not very useful in clinical settings as a tool for evaluation or management. Some researchers have calculated cMetS-S using traditional MetS components’ values to overcome these limitations (10-12). The cMetS-S not only detects individuals with MetS but also provides cardiometabolic insights for those without MetS (10). Furthermore, it enables the comparative assessment of associated health risks across both groups.

Many studies have reported the correlation between MetS and both CVD and CVD mortality (1). A meta-analysis study in Japan revealed that MetS significantly increased the risk of CVD morbidity with HR (95% CI) of 1.71 (1.34 - 2.18) and 1.89 (1.45 - 2.46) for men and women, respectively, and the risk of CVD mortality with HR (95% CI) of 1.68 (1.37 - 2.06) and 1.73 (1.39 - 2.15) for men and women, respectively (24). Ramezankhani et al. investigated how changes in MetS status and its components were related to the risk of CVD and compared these relations in women versus men. Their findings indicated an independent relation between MetS and an increased risk of CVD in both genders. The associations were stronger in women with HR (95% CI) of 2.76 (2.00 - 3.82) than in men with HR (95% CI) of 1.60 (1.23 - 2.09) for CVD (25).

Regarding the association of cMetS-S, a few studies have assessed the relation of cMetS-S with CVD and mortality. In a study conducted by Honarvar et al., cMetS-S had a strong correlation with CVD and all-cause mortality with an HR (95%CI) of 1.67 (1.47 - 1.89) and 1.37 (1.11 - 1.69) per 1 - SD increase in cMetS-S, respectively (6). The Kailuan cohort study revealed a consistent increase in the risk of CVD and all-cause mortality as the MetS score increased. The HRs (95% CI) for CVD and all-cause mortality were 2.05 (1.86 - 2.25) and 1.45 (1.35 - 1.56), respectively, in individuals above the 75th percentile compared to those below the 25th percentile (9). Also, a recent cohort study in Chinese adults showed that the MetS severity score was strongly related to CVD risk. Compared with the lowest quartile (Q1) of the MetS severity score, the HRs (95% CI) for CVD in the Q2, Q3, and Q4 were 1.812 (1.329 - 2.470), 1.746 (1.265 - 2.410), and 2.817 (2.015 - 3.938), respectively (26). Jang et al. showed that the MetS score among fairly healthy middle-aged Korean adults could predict future CVD better (AUC: 0.72) than the traditional MetS definition (AUC: 0.718) using National Adult Treatment Panel III (ATPIII) criteria (the presence of at least three of MetS components) and demonstrated that CVD risk was gradually higher in the higher MetS score quartile compared to the lowest MetS score quartile (15). However, a cut-off point for cMetS-S for CVD and CVD mortality has not been established.

Lower cut-off points for cMetS-S in women may be justified by sex disparities in the impact of MetS and its components on CVD and CVD mortality, with distinct mechanisms influencing outcomes in men and women. Research indicates that while men generally exhibit a higher prevalence of metabolic risk factors, women experience more severe consequences from certain components of MetS, such as low HDL cholesterol and high fasting glycemia, which are linked to increased mortality rates. These sex differences may be due to various factors, including sex hormones, body composition, and differences in the experience and response to MetS (27-29).

In the current study, SBP was more strongly associated with the risk of CVD and CVD mortality than other MetS components. Additionally, SBP exhibited even greater AUC and predictive power for CVD and CVD mortality compared to cMetS-S. The higher association of SBP than other MetS components with CVD and CVD mortality has been reported previously (1, 30). Furthermore, SBP was reported to be a better predictive factor for CVD than MetS as a whole entity (30). The comparative advantage of cMetS-S over SBP for predicting CVD and CVD mortality requires further studies.

This study has several strengths. First, the prospective cohort was well designed, with a large sample of Iranian adults and a long follow - up period of 18 years. Furthermore, this is the first study to determine the cut-off point of cMetS-S to use as an index for predicting CVD and CVD mortality, which makes it a more clinically applicable health metric. Some limitations should also be acknowledged. First, this study was carried out with a group of people living in Tehran, so our results might not be generalized to the entire country or other populations. It is also challenging to establish the cut-off point for cMetS-S in other age groups and races.

To sum up, the cMetS-S cut-off points differed between men and women and had better SS and SP for predicting CVD and CVD mortality than MetS in the Iranian population. This can assist physicians in screening and managing individuals at high risk. Moreover, it is essential to conduct multicenter studies to confirm the results presented in the existing literature.

## supplementary material

ijem-22-4-154255-s001.pdf

## Data Availability

The dataset presented in the study is available on request from the corresponding author during submission or after its publication. The data are not publicly available due to ethical legal and commercial restrictions.
