# Supplementary material for: Optimal Cut-off Points of the Standardized Continuous Metabolic Syndrome Severity Score (cMetS-S) for Predicting Cardiovascular Disease (CVD) and CVD Mortality in the Tehran Lipid and Glucose Study (TLGS)
Source: Int J Endocrinol Metab. 2024 Dec 18;22(4):e154255. doi: 10.5812/ijem-154255 (PMC11892520; doi:10.5812/ijem-154255)
Supplement: ijem-22-4-154255-s001.pdf [file ijem-22-4-154255-s001.pdf]

Appendix 1. Age- and sex-specific equations of continuous metabolic syndrome severity score (cMetS-S) using confirmatory factor analysis

Sex	Age	Equations
Male	20 – 39 yr.	-1.79 + 0.0016 × SBP + 0.0045 × WC + 0.0017 × FPG + 0.24 × ln (TG) - 0.0042× HDL-C
	40 – 60 yr.	-1.67 + 0.0007 × SBP + 0.0034 × WC + 0.0014 × FPG + 0.25 × ln (TG) - 0.0042× HDL-C
Female	20 – 39 yr.	-2.43 + 0.0039 × SBP + 0.0066 × WC + 0.004 × FPG + 0.28 × ln (TG) - 0.0052 × HDL-C
	40 – 60 yr.	-2.37 + 0.001 × SBP + 0.0021 × WC + 0.0015 × FPG + 0.41 × ln (TG) - 0.004 × HDL-C

Abbreviations: SBP, systolic blood pressure; WC, waist circumference; FPG, fasting plasma glucose; TG, triglyceride; HDL-C, high-density lipoprotein cholesterol

Appendix 2. Study population's baseline characteristics, according to metabolic syndrome severity score quartiles

Characteristics	Q1	Q2	Q3	Q4	P-value
Number of participants					
Male, n (%)	660 (33.95)	878 (45.16)	902 (46.40)	963 (49.54)	
Age years, mean ± SD	42.69 ±11.86	46.05 ±12.03	47.91 ±12.03	50.73 ±11.74	<.001
BMI (kg/m2), mean ± SD	24.63 ±3.94	26.98 ±3.96	28.55 ±4.07	29.84 ±4.37	<.001
Waist circumference (cm), mean ± SD	80.83 ±9.23	88.72 ±9.27	94.05 ±8.94	98.65 ±9.69	<.001
Education, n (%)					<.001
Illiterate/primary school (<6 yrs.)	1173 (63.58)	1206 (67.19)	1193 (67.17)	1204 (70.74)	
High school (6-12 years)	396 (21.46)	359 (20.00)	370 (20.83)	303 (17.80)	
Higher education (>12 years)	276 (14.96)	230 (12.81)	213 (11.99)	195 (11.46)	
Current Smoking, n (%)	270 (13.92)	307 (15.81)	281 (14.48)	286 (14.73)	<.001
Physical activity, n (%)					<.001
Low	517 (26.75)	500 (25.84)	473 (24.46)	486 (25.09)	
Moderate	326 (16.86)	325 (16.80)	323 (16.70)	286 (14.77)	
High	1090 (56.39)	1110 (57.36)	1138 (58.84)	1165 (60.14)	
Family history of CVD	303 (15.59)	305 (15.71)	340 (17.50)	336 (17.30)	<.001
SBP (mmHg), mean ± SD	110.86 ±14.40	117.97 ±16.81	124.13 ±18.79	132.24 ±21.77	<.001
DBP (mmHg), mean ± SD	73.34 ±9.55	77.21 ±9.85	80.56 ±10.52	83.53 ±10.95	<.001
FBS (mg/dL), mean ± SD	87.91 ±11.04	91.92 ±14.97	96.14 ±20.71	126.15 ±56.77	<.001
Triglyceride (mg/dL), mean ± SD	87.20 ±24.54	137.97 ±33.15	190.10 ±47.28	313.87 ±155.70	<.001
Total cholesterol (mg/dL), mean ± SD	191.24 ±37.31	210.31 ±39.61	220.02 ±42.54	236.24 ±48.84	
HDL-C (mg/dL), mean ± SD	49.39 ±11.48	42.59 ±9.56	38.88 ±8.68	35.41 ±8.43	<.001
Anti-hypertensive drug use, n (%)	37 (2.91)	80 (6.28)	142 (10.81)	234 (18.03)	<.001
Anti-diabetic drug use, n (%)	15 (0.77)	24 (1.24)	51 (2.62)	243 (12.51)	<.001
Lipid-lowering drug use, n (%)	9 (0.46)	23 (1.18)	49 (2.52)	128 (6.59)	<.001
cMetS-S, mean ± SD	-1.25 ±0.45	-0.33 ±0.19	0.30 ±0.18	1.28 ±0.57	<.001

BMI: Body mass index, CVD: Cardiovascular disease, DBP: Diastolic blood pressure, SBP: Systolic blood pressure, HDL-C: High density lipoprotein cholesterol, FBS: Fasting blood sugar, n: Number, SD: Standard deviation
